# Engagement in Digital Health App-Based Prevention Programs Is Associated With Weight Loss Among Adults Age 65+

**DOI:** 10.3389/fdgth.2022.886783

**Published:** 2022-05-19

**Authors:** Lisa A. Auster-Gussman, Kimberly G. Lockwood, Sarah A. Graham, Viveka Pitter, OraLee H. Branch

**Affiliations:** Lark Health, Mountain View, CA, United States

**Keywords:** older adults, mHealth, weight loss, prevention, engagement, digital health, diabetes

## Abstract

**Background:**

Digital health programs have been shown to be feasible and effective for the prevention of chronic diseases such as diabetes. Contrary to expectations, findings also suggest that older adults have higher levels of engagement with digital health programs than younger adults. However, there is a paucity of research examining outcomes among older adults in digital health programs and whether higher engagement is related to better outcomes.

**Methods:**

We examined weight loss outcomes for 538 users aged 65 and older participating in one of two app-based prevention programs called the Diabetes Prevention Program and the Prevention Program, respectively. Both programs were available on a single artificial intelligence (AI)-powered digital health platform and shared a common goal of weight loss. We also examined the relationship between key engagement metrics (i.e., conversing with the AI-powered coach, weigh-ins, and initiating educational lessons early in the program) and weight loss outcomes.

**Results:**

The average weight loss of all enrollees having a weight measurement after after the 9th week was 4.51%, and the average weight loss of the Diabetes Prevention Program enrollees meeting a minimum engagement level was 8.56%. Greater weight loss was associated with a greater number of days with AI-powered coaching conversations (*p* = 0.03), more weigh-ins (*p* = 0.00), and early educational lesson initiation (*p* = 0.02).

**Conclusions:**

Digital health programs powered by AI offer a promising solution for health management among older adults. The results show positive health outcomes using app-based prevention programs, and all three engagement metrics were independently associated with weight loss.

## Introduction

The population of older adults, 65 years and older, in the United States is increasing rapidly (1). As a result, a top healthcare priority is the expansion and improvement of programs for prevention of chronic diseases, which are especially prevalent among older adults (1). Scalable solutions for disease prevention are essential given that there are over 54 million older adults in the United States as of 2020 and both their numbers and risk for chronic diseases, such as diabetes, hypertension, and obesity, are increasing (1). Digital health programs are a scalable way to facilitate the prevention of chronic diseases across age groups, and emerging research suggests that this includes older adults (2).

Although past researchers have deemed age to be the “largest barrier to digital health adoption” (3), recent evidence suggests older adults are not only willing to engage with digital health offerings but do so at similar or even higher rates than younger adults. For example, older adults report willingness to engage with smartphone-based technologies for pain management (4). Moreover, recent research indicates that older adults show higher engagement with digital health programs than younger adults, contrary to conventional wisdom that older adults face too many barriers to engage with digital health programs at the same level as younger adults (5). The results from Graham and colleagues (5), comparing engagement in digital health programs of adults 35 to 64 years to those over 65, demonstrated that adults over 65 engaged in significantly more coaching conversations, logged more meals, and provided more connected device measurements than younger adults. Furthermore, research on engagement in a digital health program among a Medicare population showed that 92% of participants completed at least nine out of 16 program lessons and engaged in 19 out of 31 opportunities for weekly program engagement (6). Among 140 participants aged 50 to 80 years, 65% showed long-term engagement, operationalized as completing at least one task activity per month over 4 months, and there were no differences when stratified by age (i.e., 50 to 64, and 65 to 80) (7). These studies provide powerful evidence that older adults are both willing and able to engage with digital health programs.

Although evidence is mounting on engagement among older adults in fully digital health programs, little research has been conducted on clinical outcomes among older adults in these programs. The published studies in this domain are primarily feasibility and acceptability trials, trials with very small samples, include adults under 65 years of age, or examine programs that are not fully digital. Qualitative acceptability research suggests that older adults believe a digital health coach may help them improve health behaviors, such as increase their physical activity, but this has not been tested (8). One study measured feasibility and acceptability of a fully digital health program among older adults and reported an average weight loss of 3.44 pounds over 4 months, showing promise for the effectiveness of fully digital health programs for this population, but only included 19 individuals (7). A meta-analysis of six trials of adults with a mean age of 68 years old indicated that programs with smartphone-based intervention components helped these individuals decrease sedentary time, increase physical activity, and increase fitness; however, these studies included adults as young as 55 years of age (9). Finally, a study using a Medicare population with a mean age of 68 years old showed weight loss at 12 months, but this study included a combination of human and digital health coaching (6). In sum, this growing literature supports the fact that older adults can both engage with and benefit from digital health offerings, but whether older adults can lose weight in a fully digital program has not been tested.

As research on engagement has mounted, questions related to the importance of the timing of engagement have emerged, with a focus on early engagement in digital health programs as a predictor of longer-term positive health outcomes. These studies were not focused on older adults, but they demonstrate the importance of early engagement, and they beg the question of whether this would be similar in the older population. For example, early program engagement predicted weight loss at 1 year in a combined behavioral and pharmacotherapy weight loss program (10). Specifically, each additional day of meal logging during the 1st 3 weeks of the program was related to a 7% increase in the odds of attaining at least 5% weight loss at 1 year (10). Research on weight loss among emerging adults in a combined web-based and in-person behavioral weight loss program showed that engagement during the initial 4 weeks of treatment, operationalized as attendance at an initial in-person session and at least weekly weight reporting, was associated with increased weight loss (11). A variety of studies have also reported the importance of early engagers in weight loss trials; specifically, those who have the best weight loss in the first month also have better long-term weight loss (12). Taken together, these studies indicate that early program engagement plays a key role in predicting later clinical program outcomes.

There is a paucity of evidence focused on outcomes specifically among older adults and on the relationship between engagement and outcomes among this population in fully digital prevention programs. Therefore, this study examined weight loss as the primary outcome of two fully digital preventive health programs because weight loss is a common metric used to assess success in such programs and because even small amounts of weight loss are related to improvements in other clinically relevant metrics such as hemoglobin A1c, triglycerides, systolic blood pressure, and LDL cholestorol (13). The primary purpose of this research was to examine weight loss among adults 65 years and older enrolled in one of two prevention programs on a single digital health platform. Specifically, we examined weight loss among this population as well as the relationship between engagement and weight loss, with a focus on the impact of early engagement. The primary hypothesis was that higher engagement would be associated with greater weight loss among older adults.

## Methods

### Study Design

This was a longitudinal, observational study of users enrolled in an AI-powered digital chronic disease prevention program available via a smartphone app called Lark. We examined weight loss as well as the relationship between engagement and weight loss. The study received exemption status from Advarra Institutional Review Board (Protocol #Pro00047181) for retrospective analyses of previously collected and de-identified data.

### Participants and Recruitment

Participants were 538 users of an AI-based chronic disease prevention coaching app who joined one of two prevention programs [see Graham and colleagues (5) for details] offered through the platform (i.e., Diabetes Prevention Program or Prevention Program), both of which had a primary outcome of weight loss. All users had private insurance and gained access to the app at no cost via partnerships between the app and their insurance provider. Those who were eligible and signed up received a link via text message to download the Lark app to their smartphones. Briefly, the Diabetes Prevention Program followed the National Diabetes Prevention Program (NDPP) guidelines and the established Prevent T2 curriculum for delaying or preventing progression to type 2 diabetes (14). Diabetes Prevention Program users must meet risk criteria established by the Centers for Disease Control and Prevention (14).

The Prevention program targets individuals who may not meet strict criteria for participation in the NDPP. The Prevention program emphasizes taking small steps that lead to significant and lasting behavior change in the areas of nutrition, physical activity, weight loss, sleep, and stress reduction. Any user with insurance coverage for Lark can sign up for the Prevention Program. Most participants in both programs (97.6% overall) had access to a connected digital body weight scale, provided through participation in the program, that automatically transmitted their weigh-ins to the digital platform.

### Inclusion Criteria

Inclusion criteria for the analytic sample were: (1) enrollment in either the Diabetes Prevention or Prevention program on or after January 1, 2019, (2) aged 65 years and older, (3) a starting BMI ≥25, (4) in the program for at least 3 months, (5) had a weight after 9 weeks, (6) had full demographic information, and (7) had weight loss set as a goal in their program. Exclusion criteria were: (1) those who had earlier versions of the app (i.e., before January 1, 2019) and (2) those who had been in the programs <3 months. We also excluded users who were normal weight or underweight because the primary outcome was weight loss.

### Program Flow

Users who qualified for the app, downloaded it, and signed up for a given program provided all measures and outcomes via the app-based digital health platform. The AI-based platform includes two prevention programs: Diabetes Prevention Program and Prevention Program. These programs include a core set of features plus condition-specific content. All programs are delivered via an iPhone or Android smartphone. They each include a series of educational lessons delivered via conversational AI as well as calls-to-action and nudges which provide positive reinforcement and prompt users to enter health data, log a meal, complete a lesson, or weigh themselves. The AI coach encourages user behavior change using cognitive behavioral therapy techniques and is available 24 hours per day for users to check in and discuss challenges or progress.

### Engagement Measures

We examined three engagement variables as predictors of weight loss and measured these engagement variables and their relation to weight loss. All predictors occurred during the first 9 weeks of the program because the focus was on modeling early engagement as a predictor of later clinical outcomes. The three engagement metrics each represented different types of engagement: conversations, early lesson initiation, and weigh-ins. *Conversations* was the percentage of days in the first 9 weeks that a user engaged in a two-way conversation within the app and represented active app usage and contact with the coach. *Early lesson initiation* was a binary variable indicating whether a user completed >2 lessons during the first 3 weeks of the program and represented engagement with educational content beyond simple contact with the coach. The *early lesson initiation* variable captured those with high early use, as a single lesson took seven days of app usage to complete. Completion of two lessons in the first 2 weeks indicated 100% daily usage, so we expanded the window to include the third week of the program. *Weigh-ins* was the number of weigh-ins during the first 9 weeks of the program and represented a real-world behavior prompted by the coach.

### Outcome Measures

The primary outcome was *percent weight loss*, calculated as (first weight-nadir weight)/first weight. First weight was the average of weights recorded on the first day a user provided weights. Nadir weight was the minimum recorded weight occurring after >9 weeks in the program, as past research suggests that the first several weeks in a program are critical to the prediction of long-term outcomes (12). As a quality control, the digital platform flags abnormal weigh-ins for review by identifying a weight loss rate of >7lbs/week and removes outliers unless confirmed by the member to be accurate. The duration of active participation in a digital health program may vary across members; thus, the use of nadir weight and time-to-nadir enabled a larger number of members to be assessed for weight loss outcomes. The mean time-to-nadir was 138 days after program enrollment (SD = 71 days), suggesting that the nadir weight occurred on average after approximately 4.5 months of program participation.

### Statistical Analyses

We conducted all statistical analyses in R version 1.4.1717 (15). Users self-reported their age, gender, race, and height upon enrollment. We calculated BMI (kg/m^2^) from height and starting weight and obtained engagement variables directly from users' interactions with the digital platform. We used linear regression to examine the association between engagement and percent weight loss (dependent variable) and log-transformed variables with non-normal distributions as necessary. We combined the Diabetes Prevention and Prevention programs for the regression since the programs shared a common goal (weight loss) and combining them increased the sample size for analysis. We also included program type as an independent variable in the model. We checked for multicollinearity of the independent variables in the model using variance inflation factors. All other analyses were simple descriptive statistics for each included variable. The *a priori* alpha level was *p* < 0.05.

## Results

### Participants

**Figure 1:**
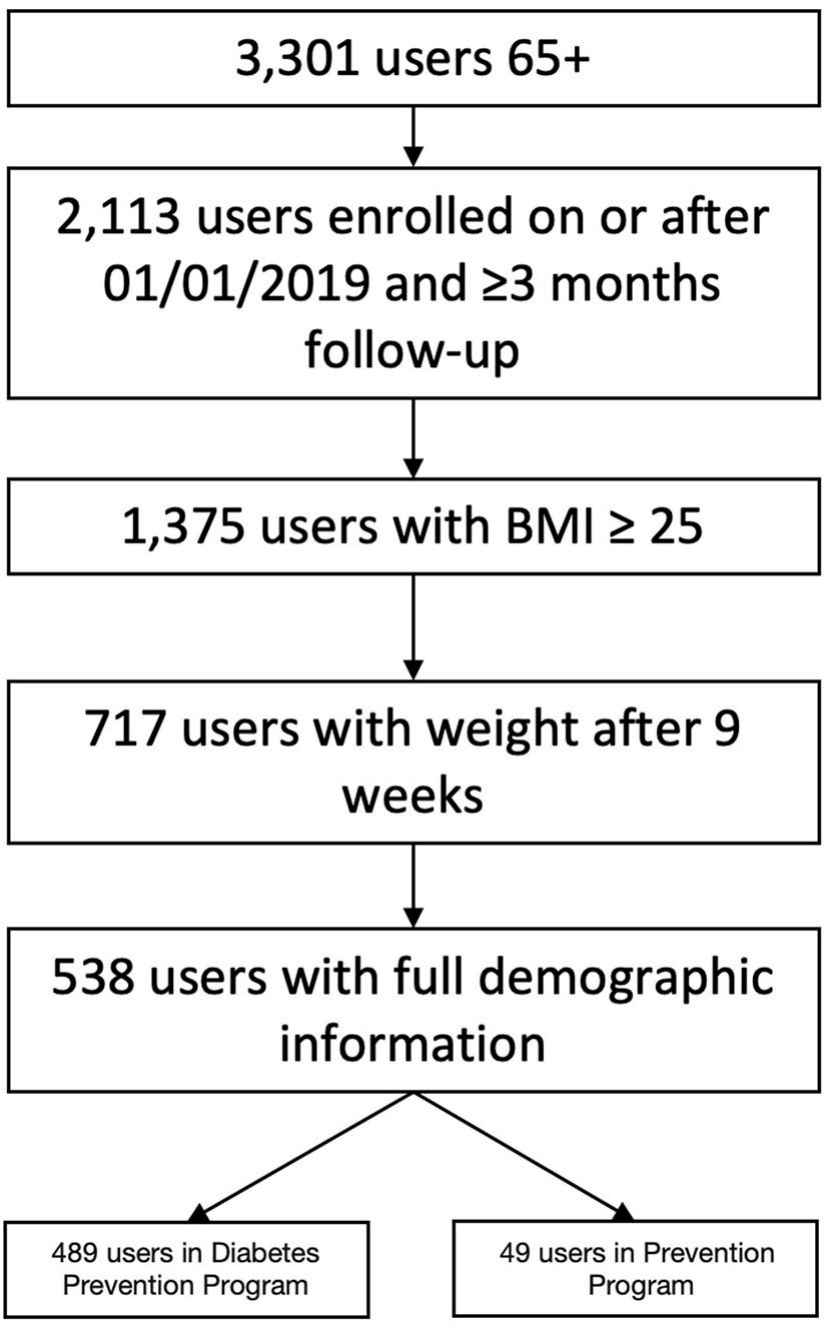
Users based on inclusion and exclusion criteria.

Our final sample included 538 users (see Figure 1 for inclusion flow chart) with most individuals enrolled in the Diabetes Prevention Program (*N* = 489). Mean age was 67.47 years (SD = 3.28). More than half of users were female (61%), and 16% were non-white. Mean Body Mass Index (BMI) was 32.61 kg/m^2^ (SD = 5.59) (see Table 1 for complete demographics and characteristics).

**Table 1:** Participants, engagement, and outcome metrics.

	**Mean (SE/CI/IQR) or % (*n*)**
**Participants**	
Age (years)	67.47 (0.14)
	Median 66.00 [25% = 65.00, 75% = 69.00]
Starting BMI (kg/m^2^)	32.61 (5.59)
	Median 31.32 [25% = 28.28, 75% = 35.64]
% Female	61% (*N* = 329)
% Non-white	16% (*N* = 84)
**Engagement features (9 weeks)**	
Mean weigh-ins	21.76 (0.80)
Mean conversations	145.58 (4.59)
% Early mission initiation	45% (*N* = 241)
**Weight loss**	
Overall (*N* = 538)	4.51% (95% CI [4.14, 4.87])
Diabetes prevention program (*N* = 489)	4.57% (95% CI [4.19, 4.94])
CDC qualifiers (*N* = 60)	8.56% (95% CI [7.03, 10.08])
Prevention program (*N* = 49)	3.91% (95% CI [2.52, 5.31])
Mean nadir weight day	137.60 (3.06)

*N = 538 unless noted otherwise; “CDC Qualifiers” indicates the subset of users in the Diabetes Prevention Program that met minimum lesson completion standards set forth by the CDC. This means completion of at least 3 lessons in the first 6 months and at least 1 lesson from months 9–12*.

### Percent Weight Loss and Engagement

Mean overall percent weight loss was 4.51% (95% CI = 4.14, 4.87). Further details and program-specific means are in Table 1. An examination of engagement revealed that the mean number of *conversations* during the first 9 weeks was 145.58 (SE = 4.59). The percentage of users with *early lesson initiation* was 45%. The mean number of *weigh-ins* during the first 9 weeks was 21.76 (SE = 0.80). The mean nadir weight day was 137.60 (SE = 3.06) days after program enrollment, suggesting that nadir weight occurred on average after approximately 4.5 months, but with variation in the time of peak loss across users.

### Relationship Between Engagement and Percent Weight Loss

The final regression model predicting percent weight loss included the effect of conversations, early lesson initiation, and weigh-ins. Control variables included user demographics as well as starting BMI, time-to-nadir weight, and program type (see Table 2 for full results; all independent variables are included in Table 2). The model had good overall fit [R^2^ = 0.3, *F*_(9, 528)_ = 24.4, *p* < 0.001]. We observed that all three engagement variables were significantly related to percent weight loss, such that a higher number of *conversations*, more *weigh-ins*, and *early lesson initiation* were related to greater weight loss. In addition, all variance inflation factor (VIF) values were between 1.03 and 1.64, indicating no issues with multicollinearity, and bivariate correlations between the engagement variables ranged from *r* = −0.006 to *r* = 0.32.

**Table 2:** Results of the regression modeling the effect of engagement on weight loss percent.

	**Unstandardized B**	**SE**	**95% CI**		***p***
			**LL**	**UL**	
**Constant**	**−8.69**	**3.63**	**−15.81**	**−1.57**	**0.02**
**Control variables**					
Sex	0.32	0.33	−0.33	0.98	0.33
Race	−0.34	0.44	−1.20	0.52	0.44
Age	0.05	0.05	−0.05	0.14	0.35
Body mass index	0.05	0.03	−0.01	0.10	0.12
**Nadir weight day**	**0.03**	**0.00**	**0.02**	**0.03**	**0.00**
**Program type** (Ref. DPP)					
Prevention	−0.11	0.55	−1.21	0.99	0.84
**Engagement**					
**Early lesson initiation**	**0.89**	**0.39**	**0.14**	**1.65**	**0.02**
**Conversations**	**0.57**	**0.25**	**0.07**	**1.07**	**0.03**
**Weigh-ins**	**0.65**	**0.19**	**0.28**	**1.01**	**0.00**

*p < 0.05 shown in bold. DPP, Diabetes Prevention Program. All independent variables used in analyses are included in table*.

## Discussion

The results of this study support the primary hypothesis that increased engagement was related to a greater percent weight loss among older adults. The relationship between program engagement and clinical outcomes suggests that digital health programs are an effective way to promote weight loss and, potentially, other related health outcomes among older adults. This research adds to the growing body of literature on older adults and digital health, further suggesting that greater engagement is associated with positive health outcomes among older adults.

### Contribution to the Literature

Contrary to past conjecture, this study supports findings from recent research that suggests older adults are able and willing to engage in digital health apps and do engage in digital health.^5^ Past research has found that adults aged 65 and older can be successful in hybrid programs (6), but this research is the first to provide evidence of success in fully digital programs on a relatively large scale. The observed average weight loss of 4.5% in this study supports a previous small-sample feasibility study of a fully digital mobile app-based program that showed an average weight loss of 3.44 pounds over 4 months, indicating that older adults can successfully lose weight using fully digital programs (7). Results from research examining the association between engagement, age, and weight loss are instructive, although they include adults across the age span from 18 to 85 years of age (16). Specifically, researchers found that the association between engagement and weight loss was stronger for younger people compared to older adults (16). Although this may be true, our findings suggest that engagement is an important predictor of outcomes among older adults; thus, there is still merit in encouraging higher engagement among older adults.

Since we found that engagement is critical for outcomes, considering how different types of engagement relate to outcomes is also important. Past research has suggested the importance of measuring engagement at different levels of analysis, namely, “Big E” and “Little e” engagement, the former referring to health behavior engagement (e.g., weigh-ins) and the latter to app engagement, which is further broken down into app-use engagement (e.g., conversations) and behavior change content engagement (e.g., educational lessons) (17). The authors suggested the importance of examining engagement at multiple levels to better understand how app-use engagement is related to outcomes (e.g., the relation between conversations and weight loss) and the ways in which the outcome is explained by health behavior engagement, which is related to but separate from app-use engagement (e.g., the unique variance contributed by weigh-ins above and beyond conversations).

Assessing engagement at multiple levels also helps researchers understand which aspects of engagement are most important for influencing a given outcome. For example, if we had found that conversations, but not early lesson completion, was significant this might indicate the primary importance of two-way interactions with coaches regardless of participation in educational content. However, in line with the suggestions of past researchers, the results of this study demonstrated that engagement at each level independently contributed to weight loss; *conversations*, which showed active app usage and contact with the coach, *early lesson initiation*, which showed engagement with educational content beyond contact with the coach, and *weigh-ins*, which showed real-world behavior prompted by the coach. Our results indicate that it is not just one type of engagement that predicts weight loss, but that greater engagement at each of these three levels uniquely contributes to this important indicator of improved health. This finding is similar to results from a study with younger adults, which showed that a cluster of different engagement variables (e.g., attendance at an initial session in person, weight reporting) predicted weight loss (11). Thus, both older and younger adults appear to benefit from multiple modes of engagement, and our results further suggest that such early multifaceted engagement predicts improved weight loss outcomes in older adults.

### Strengths and Limitations

There were several limitations in this study. All data were retrospective and collected via the digital health app platform. There were fewer users in the Prevention Program compared to the Diabetes Prevention Program, but the regression coefficient for program was not significant indicating that weight loss did not differ by program. The study only included users who had full demographic data available, as well as a BMI ≥25 and a weight loss goal, which led to a decrease in the available sample size. However, including complete data on all variables was an important first step in revealing predictors of weight loss among older adult users of a fully digital program. We aimed to capture engagement at three different levels, namely app-use engagement, behavior change content engagement, and health behavior (i.e., weigh-in) engagement. These measures provided important engagement insights but were still relatively rudimentary; future research could explore more nuanced types of engagement (e.g., patterns of engagement over time) or ways of operationalizing engagement that better capture unique user behavior patterns or trends. In line with research suggesting that older adult internet usage is increasing (18), these findings suggest that the digital divide may not be as wide as it once was. However, we only examined older adults who were willing and able to sign up for a digital app. Future research could examine whether there are age-specific factors in digital health design or content that lead to greater willingness of older adults to use fully digital health programs or facilitate even greater engagement given that encouraging and maintaining high rates of engagement is a key challenge in digital health. The primary strength of this study was that all data came from real-world users of the app rather than participants recruited specifically for a research study. Future users of the app would have the same user experience as this sample since there were no study components that occurred outside of the app interface.

## Conclusions

The findings of this study suggest that older adults lose weight while using preventive health programs on a fully digital platform, and that increased engagement is related to increased weight loss. As the need for scalable solutions for older adults rapidly increases over the coming years, digital health programs may offer a solution to the rapidly increasing needs of individuals and communities at risk for, and living with, chronic diseases. Scalable, fully digital health solutions were previously thought to be efficacious only among younger adults. However, the findings presented here reveal that this assumption is, at the very least, worthy of being tested in research. These findings are a first step in developing a body of literature supporting digital health solutions for all ages, including older adults.

## Data Availability Statement

The raw data supporting the conclusions of this article will be made available by the authors, without undue reservation.

## Ethics Statement

The studies involving human participants were reviewed and approved by Advarra (Protocol #Pro00047181) Institutional Review Board. Written informed consent for participation was not required for this study in accordance with the national legislation and the institutional requirements.

## Author Contributions

VP conducted data cleaning, organization, and analysis for this manuscript. LA-G conducted data analysis. KL, SG, and OB assisted in analysis, writing, and editing. All authors contributed to the article and approved the submitted version.

## Conflict of Interest

All authors are employed by Lark Health.

## Publisher's Note

All claims expressed in this article are solely those of the authors and do not necessarily represent those of their affiliated organizations, or those of the publisher, the editors and the reviewers. Any product that may be evaluated in this article, or claim that may be made by its manufacturer, is not guaranteed or endorsed by the publisher.
